# Supplementary material for: British Columbia’s Experience after Implementation of the Treponema pallidum Reverse Algorithm and PCR Detection, 2015 to 2020
Source: Microbiol Spectr. 2022 Jun 6;10(3):e00686-22. doi: 10.1128/spectrum.00686-22 (PMC9241594; doi:10.1128/spectrum.00686-22)
Supplement: Supplemental file 1 — Supplemental material. Download spectrum.00686-22-s0001.pdf, PDF file, 0.1 MB [file spectrum.00686-22-s0001.pdf]

Supplementary Table 1. Expected RFLP fragment patterns for azithromycin resistance testing

| Mutation | Panel 1* fragment size | Panel 2** fragment size |
|---|---|---|
| None | 629 bp | 629 bp |
| A2058G | 449 bp +180 bp | 629 bp |
| A2059G | 629 bp | 436 bp + 193 bp |
| A2058G + A2059G | 429 bp + 30 bp + 170 bp | 436 bp + 193 bp |

*Panel 1 enzymes: *Mbo*II and *Bsa*XI targeted to A2058G

**Panel 2 enzyme: *Bsa*I targeted to A2059G

Abbreviations: RFLP=Restriction Fragment Length Polymorphism
