# Supplementary material for: British Columbia’s Experience after Implementation of the Treponema pallidum Reverse Algorithm and PCR Detection, 2015 to 2020
Source: Microbiol Spectr. 2022 Jun 6;10(3):e00686-22. doi: 10.1128/spectrum.00686-22 (PMC9241594; doi:10.1128/spectrum.00686-22)
Supplement: Supplemental file 2 — Supplemental material. Download spectrum.00686-22-s0002.pdf, PDF file, 0.1 MB [file spectrum.00686-22-s0002.pdf]

Supplementary Table 1. In-house syphilis PCR primers

	Name	Oligo Sequence (5' → 3')
Forward Primer	TP47_TQ1_F	GGATAGTTTTTCTGCACGTAAGGTAA
Forward Primer	TP47_TQ1_F2	CTGAGGATAGTTTTTCTGCACGTAAGGTAA
Reverse Primer	TP47_TQ1_R	ACCCACCGTGTCTACCACAAG
Probe	TP47_TQ1_P	6FAM-CAG CAT GGA GAG CCC GCA CG-MGBNFQ

	Name	Oligo Sequence (5'→ 3')
Forward Primer	TPPA_TQ_F	GGTAGAAGGGAGGGCTAGTA
Reverse Primer	TPPA_TQ_R	CTAAGATCTCTATTTTCTATAGGTATGGCA
Reverse Primer	TPPA_TQ_R2	CTAAGATCTCTATTTTCTATAGGTATGGCG
Probe	TPPA_TQ_P	VIC-AGACACAGCACTCGTCTTCAACTCCGACT-MGBNFQ

Supplementary Table 2. Expected RFLP fragment patterns for azithromycin resistance testing

Mutation	Panel 1* fragment size	Panel 2** fragment size
None	629 bp	629 bp
A2058G	449 bp +180 bp	629 bp
A2059G	629 bp	436 bp + 193 bp
A2058G + A2059G	429 bp + 30 bp + 170 bp	436 bp + 193 bp

*Panel 1 enzymes: *Mbo*II and *Bsa*XI targeted to A2058G

**Panel 2 enzyme: *Bsa*I targeted to A2059G

Abbreviations: RFLP=Restriction Fragment Length Polymorphism

Supplementary Material: BC Centre for Disease Control Syphilis Case Definition

Primary Syphilis Confirmed Case:

Clinical presentation of one or more ulcers or chancre;

AND

Presence of one of the following:

- detection and confirmation of Treponema pallidum in clinical specimens (e.g., chancre, regional lymph node) by appropriate laboratory techniques, such as dark-field microscopy, direct fluorescent antibody, or nucleic acid amplification test (NAAT); OR

- reactive treponemal (Note 2) serology (regardless of non-treponemal serology reactivity) in a case with no previous history of syphilis; OR

- four-fold or greater increase (e.g., 1:8 to 1:32) in titre over the last known non-treponemal test (Note 3).

Secondary Syphilis Confirmed Case:

Clinical presentation of rash, fever, malaise, lymphadenopathy, mucous lesions, condyloma latum, alopecia, meningitis, headaches, uveitis, retinitis, or recent hearing impairment;

AND

Presence of one of the following:

- detection and confirmation of T. pallidum in clinical specimens (e.g., chancre, regional lymph node) by appropriate laboratory techniques, such as dark-field microscopy, direct fluorescent antibody, or nucleic acid amplification test (NAAT); OR

- reactive treponemal serology (regardless of non-treponemal serology reactivity) in a case with no previous history of syphilis; OR

- four-fold or greater increase in titre over the last known non-treponemal test.

Note: The possibility of a prozone reaction should be considered in individuals who are suspected of having secondary syphilis but whose non-treponemal test is non-reactive. Neurological symptoms may be present.

Early Latent Syphilis Confirmed Case:

No signs or symptoms of primary or secondary syphilis and either reactive treponemal serology (regardless of non-treponemal serology reactivity) or four-fold or greater increase in titre over the last known non-treponemal test;

AND

Presence of one of the following within the previous 12 months:

- non-reactive serology; OR

- signs or symptoms suggestive of primary or secondary syphilis; OR

- sexual exposure to a partner with primary, secondary, or early latent syphilis.

Early Latent Syphilis Probable Case:

No signs or symptoms of primary or secondary syphilis and either reactive treponemal serology (regardless of non-treponemal serology reactivity) or four-fold or greater increase in titre over the last known non-treponemal test;

AND

Presence of one of the following within the previous 12 months:

- a titre at least or greater than 1:8 at time of diagnosis; OR

- is a member of a group at known increased risk of acquiring syphilis in British Columbia; OR

- sexual exposure to a partner, in the previous 12 months, who is a member of a group at known increased risk of acquiring syphilis in British Columbia.

Late Latent Syphilis Confirmed Case:

No signs or symptoms of primary or secondary syphilis;

AND

Persistently reactive treponemal serology (regardless of non-treponemal serology reactivity); AND

Does not meet the case definition for early latent (confirmed or probable) syphilis; AND

Has not been previously treated for syphilis.

Neurosyphilis Confirmed Case:

Meets the case definition for any stage of syphilis; OR

Reactive treponemal serology (regardless of non-treponemal serology reactivity);

AND

Presence of one of the following:

- reactive VDRL in non-bloody cerebrospinal fluid (CSF); OR

- clinical evidence of neurosyphilis and either elevated CSF leukocytes or elevated CSF protein in the absence of other known causes.

Neurosyphilis Probable Case:

Meets the case definition for any stage of syphilis; OR

Reactive treponemal serology (regardless of non-treponemal serology reactivity)

AND

Clinical diagnosis of neurosyphilis, based on compatible clinical or laboratory findings, in the absence of other known causes.

Early (infectious) neurosyphilis – diagnosis of neurosyphilis within 12 months after infection (i.e., neurosyphilis coincides with primary, secondary or early latent syphilis)

Late (non-infectious) neurosyphilis – diagnosis of neurosyphilis more than 12 months after infection

Ocular Syphilis Confirmed Case:

Meets the case definition for any stage of syphilis; OR

Reactive treponemal serology (regardless of non-treponemal serology reactivity);

AND

Clinical presentation of uveitis, retinitis or optic neuropathy in the absence of other known causes.

Tertiary Syphilis Confirmed Case:

Reactive treponemal serology (regardless of non-treponemal serology reactivity); AND

Characteristic abnormalities of the cardiovascular system, bone, skin or other tissue (Note 4), in the absence of other known causes.

Congenital Syphilis Confirmed Case:

Clinical presentation (Note 5) compatible with congenital syphilis in a stillbirth, neonate or older case;

AND

Presence of one of the following:

- titre greater than maternal titre and reactive treponemal confirmatory test; OR

- detection and confirmation of T. pallidum in clinical specimens (e.g., lesions, placenta, umbilical cord, or autopsy) by appropriate laboratory techniques, such as dark-field microscopy, direct fluorescent antibody assay or polymerase chain reaction (PCR); OR

- mother with untreated or inadequately treated syphilis (i.e., primary, secondary, early latent, or late latent syphilis) during pregnancy or at birth.

Early congenital syphilis – onset less than two years of age, including stillbirth

Late congenital syphilis – onset at two or more years of age

Maternal Syphilis Confirmed Case:

Meets the case definition for infectious syphilis (i.e., primary, secondary or early latent) or late latent syphilis;

AND

Presence of one of the following:

- syphilis serology conducted as part of prenatal blood screening; OR

- known to have given birth to an infant, live or stillborn, with congenital syphilis; OR

- clinical presentation of infectious syphilis during pregnancy.

Syphilis Stage Unspecified:

Reactive treponemal serology (regardless of non-treponemal serology reactivity) in an individual either with no previous history of syphilis or four-fold or greater increase in titre over the last known non-treponemal test; AND

Follow-up to determine staging of syphilis is not complete.

Notes:

1. Syphilis is a complex sexually transmitted infection that has a highly variable clinical course. Classification by a clinician with expertise in syphilis may take precedence over the above case definitions developed for surveillance purposes.
2. Treponemal test includes TP-PA, EIA, CIA or equivalent serologic methods.
3. Non-treponemal test includes VDRL, RPR or equivalent serologic methods.
4. T. pallidum is rarely seen in these lesions and is diagnostic when present although not required to meet the surveillance case definition.
5. Clinical presentation includes any evidence of congenital syphilis on physical examination (e.g., skin lesions, lymphadenopathy, hepatosplenomegaly); on radiographs of long bones; a reactive CSF-VDRL; or an elevated CSF cell count or protein in the absence of other known causes. Note that neonates may not display clinical manifestations of congenital syphilis thus may only meet laboratory criteria.
